# Supplementary material for: How Nonlinear-Type Time-Frequency Analysis Can Help in Sensing Instantaneous Heart Rate and Instantaneous Respiratory Rate from Photoplethysmography in a Reliable Way
Source: Front Physiol. 2017 Sep 22;8:701. doi: 10.3389/fphys.2017.00701 (PMC5615790; doi:10.3389/fphys.2017.00701)
Supplement: Supplementary file 1 [file Presentation1.pdf]

Online Supplementary Information for

How bluenonlinear-type time-frequency analysis can help in sensing instantaneous heart rate and instantaneous respiratory rate from photoplethysmography in a reliable way

by A. Cicone and H.-T. Wu

1 MORE DETAILS ABOUT THE RESULTS

1.1 Capnobase benchmark database – PPG signal with respiration

As is shown in Table 1 in the main article, the performance of the deppG method is better than other methods proposed in the literature. To better understand such performance, we report also the boxplots in Figure SI.1.

The Capnobase benchmark database provides labels regarding intervals containing potential artifacts in the PPG, ECG and capnometry signals. Other methods proposed in the literature, to the best of our knowledge, always exploit this information to remove these intervals in computing the statistics. However, it is important to note that to address the need for automatic annotation of respiratory and heart rates in a real scenario, the performance of deppG is obtained disregarding all the information about the intervals containing potential artifacts: we do use all the 42 datasets in the Capnobase benchmark database and all their intervals, even the ones known to contain artifacts.

On the other hand, the reference curves provided in the database may not be reliable in the intervals containing such artifacts. For instance in Figure SI.2 we report for the dataset `0329_8min` the ECG signal, the de-shaped spectrogram as well as the reference and extracted curves corresponding to IHR. Here, it is evident that the reference AHR curve, which is marked in solid red, has been produced using some kind of interpolation for the interval 244 - 410 seconds that has been reported as containing artifacts.

In order to have a better comparison with the performance of other methods, we recompute the root mean square (RMS) error and mean absolute error (MAE) of the proposed deppG and deppG-60s methods, when points inside the intervals containing artifacts are not taken into account. The results are shown in Table SI.1 as “artifacts removed”. If we compare these statistics with the ones obtained without removing the artifact intervals, we observe that for the HR, we have better performance in the instantaneous case, while the performance does not change in the average case. Regarding the respiratory rate, instead, there are no changes at all in the performance both for the instantaneous and average cases.

To better understand this phenomenon, we take a close look at the 42 datasets and their intervals containing artifacts. We discover that there are four datasets for which the beginning of expiration values are missing in one or more intervals which have not been labeled as containing artifacts. An example is shown in Figure SI.3, where the PPG signal and the capnometry for `0032_8min` is reported. On the other hand, the artificial intervals of the ECG signal are all well labeled. We provide complete names of these datasets and the corresponding intervals, denoted using sample point positions of their boundaries:

`0031_8min` [1, 38665], [39644, 67316], [71295, 76682], [83126, 144001],

`0032_8min` [85445, 95970],

`0328_8min` [116923, 124135],

`0370_8min` [15271, 24817], [46804, 78243].

If we also take into account these new intervals in removing artifacts for the computation of the deppG method performance, we obtain the statistics shown in Table SI.1 as “artifacts removed, updated”. As expected, the performance for the HR estimation does not change. However, for the RR we have a slight improvement. This extra performance evaluation indicates the importance of taking the signal quality index (SQI) [5, 4] into account. For any practical application, it is clear that, in general, we do not have

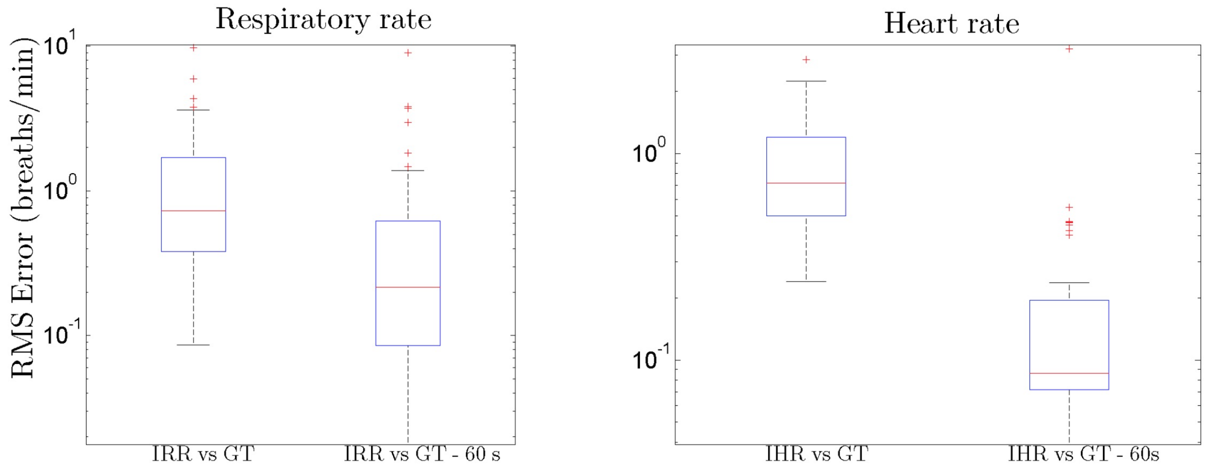

Figure SI.1. The boxplots of the root mean square error (RMS) of the instantaneous respiratory and the instantaneous heart rate estimation are shown on the left and right panel respectively. They have been generated using the Matlab function `boxplot`. The boxplots include the RMS of the instantaneous values or the average value over a 60 seconds window versus the given reference or ground truth (GT). The first, second, and third quartiles are displayed as bottom, middle, and top horizontal line of the boxes. Whiskers represents the most extreme values within three times the interquartile range from the quartile. Crosses represents outliers.

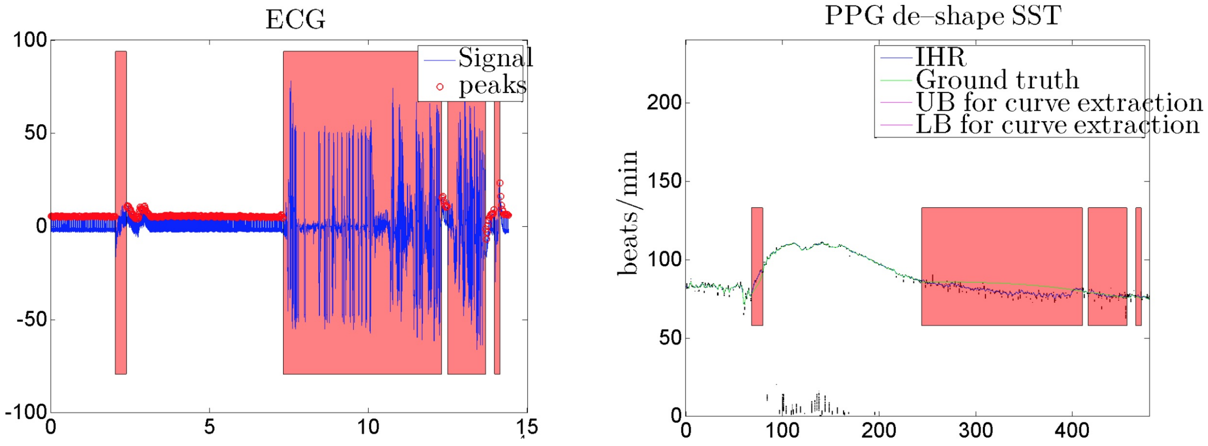

Figure SI.2. Dataset `0329_8min`. The electrocardiogram (ECG) signal is shown on the left panel. Whereas, on the right panel, it is shown the de-shaped spectrogram of the PPG signal that is superimposed with the reference averaged heart rate and the extracted curve corresponding to the instantaneous heart rate. The red boxes highlight the intervals reported in the database as containing artifacts. The reference curve is plotted in solid red. LB: lower boundary; UB: upper boundary.

any ground truth and experts are not available to determine which interval is of low quality. We could than apply the SQI designed for the PPG signal, the ECG signal, and so on, to determine which interval can be trusted. Since the SQI is not the focus of this paper, we plan to report the research result by taking the SQI into account in the deppG application to a signal in a future work.

Another important aspect to point out is that the reference curves provided are intended to be used for estimating AHR and ARR over a time window, whereas the proposed deppG method allows the computation of the *instantaneous* respiratory and heart rates. In order to ensure that such curves are also reliable for evaluating the instantaneous performance of the proposed deppG method, we want to compute new instantaneous reference curves. We compute them using expiration beginnings and R-peaks position, which are also provided in these datasets. In particular, in each time instant in the middle of two consecutive expirations in the capnogram signal (respectively two consecutive R-peaks in the ECG signal),

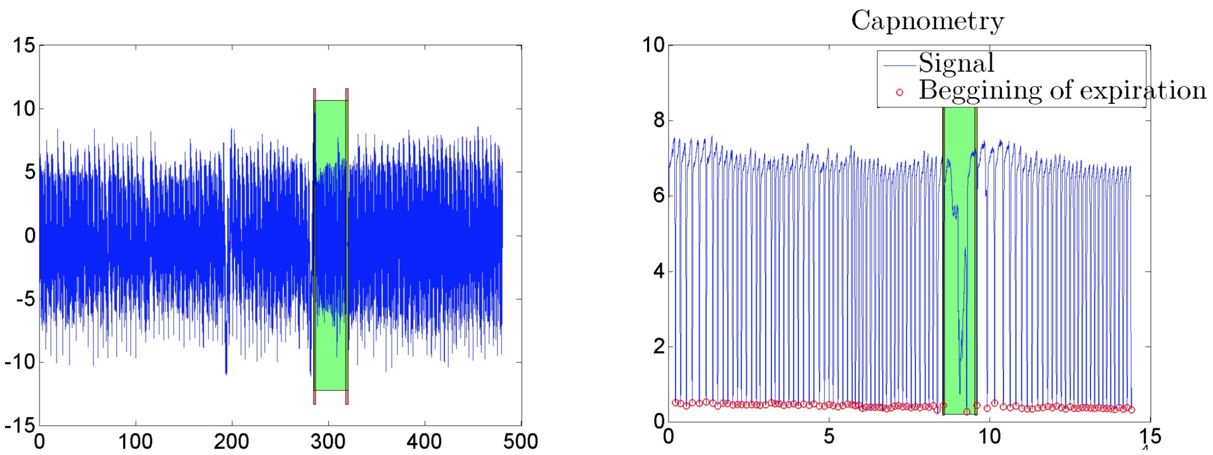

Figure SI.3. The PPG signal of the dataset `0032_8min` is shown on the left, and the simultaneously recorded capnometry signal is shown on the right. Red boxes highlight intervals reported in the database as containing artifacts. The green box highlights the newly identified interval in the capnometry containing artifacts.

Given Reference	HR (beats/minute)	RMS					MAE				
		mean	std	Q_1	median	Q_3	mean	std	Q_1	median	Q_3
deppG	whole database	0.93	0.57	0.50	0.72	1.20	0.61	0.35	0.38	0.52	0.84
	artifacts removed	0.79	0.41	0.50	0.67	1.06	0.55	0.27	0.37	0.48	0.76
	artifacts removed, updated	0.79	0.43	0.46	0.67	1.06	0.56	0.28	0.36	0.48	0.77
deppG-60s	whole database	0.23	0.49	0.07	0.09	0.19	0.15	0.29	0.05	0.07	0.12
	artifacts removed	0.24	0.54	0.07	0.09	0.19	0.16	0.32	0.05	0.07	0.12
	artifacts removed, updated	0.24	0.54	0.07	0.09	0.19	0.16	0.32	0.05	0.07	0.12

Given Reference	RR (breaths/minute)	RMS					MAE				
		mean	std	Q_1	median	Q_3	mean	std	Q_1	median	Q_3
deppG	whole database	1.39	1.87	0.38	0.73	1.70	0.94	1.37	0.22	0.50	0.83
	artifacts removed	1.39	1.88	0.38	0.73	1.70	0.94	1.39	0.22	0.50	0.83
	artifacts removed, updated	1.37	1.90	0.38	0.73	1.70	0.91	1.33	0.22	0.50	0.83
deppG-60s	whole database	0.78	1.60	0.09	0.22	0.62	0.53	1.16	0.07	0.15	0.43
	artifacts removed	0.78	1.60	0.09	0.22	0.62	0.53	1.16	0.07	0.15	0.43
	artifacts removed, updated	0.81	1.74	0.09	0.22	0.62	0.56	1.32	0.07	0.15	0.43

Table SI.1. The root mean square error (RMS) and mean absolute error (MAE) of the respiratory rate (RR) and heart rate (HR) estimation obtained by the deppG method for the Capnobase benchmark database in the following three scenarios. The estimated RR and HR are compared with the reference curves provided in the database. In the lines labeled “whole database” we provide performance of the methods based on the whole database without removing any interval. The results are the same as those shown in Table 1 in the main article. Second, in the lines labeled “artifacts removed”, we provide results when skipping labeled intervals containing artifacts provided in the database. Third, in the lines labeled “artifacts removed, updated” we provide performance when we further skip intervals for which no beginning of expiration information are provided, in addition to skipping intervals labeled as containing artifacts. The unit of the HR is beats per minute, whereas the unit of RR is breaths per minute. Std: standard deviation. Q_1 and Q_3: first and third quartile.

we compute the reciprocal of the length of the time interval between these two expirations (respectively R-peaks). We then derive the IRR (respectively IHR) as the cubic spline interpolation of such values. In Table SI.2, we report the performance of the deppG method computed using the newly computed IRR and IHR as reference curves.

Regarding the performance of the deppG method when we use the newly generated reference curves, it has to be taken into account that in some datasets the R-peaks and expiration beginnings positions are not provided for the entire signal. Nevertheless, we compute the new reference curves without making any assumption on the datasets and using the provided R-peaks and expiration beginnings as if they were

New Reference	HR (beats/minute)	RMS mean	RMS std	RMS Q_1	RMS median	RMS Q_3	MAE mean	MAE std	MAE Q_1	MAE median	MAE Q_3
deppG	whole database	2.76	5.59	0.76	0.97	1.56	1.38	3.12	0.54	0.69	1.10
	artifacts removed	1.01	0.56	0.67	0.85	1.28	0.72	0.34	0.52	0.64	0.92
	artifacts removed, updated	1.02	0.58	0.67	0.85	1.28	0.72	0.35	0.52	0.64	0.92
deppG-60s	whole database	1.16	4.42	0.05	0.07	0.25	0.71	2.87	0.05	0.06	0.17
	artifacts removed	0.82	2.36	0.05	0.07	0.25	0.49	1.50	0.05	0.06	0.17
	artifacts removed, updated	0.82	2.36	0.05	0.07	0.28	0.50	1.50	0.05	0.06	0.19

New Reference	RR (breaths/minute)	RMS mean	RMS std	RMS Q_1	RMS median	RMS Q_3	MAE mean	MAE std	MAE Q_1	MAE median	MAE Q_3
deppG	whole database	2.42	9.14	0.28	0.54	1.16	1.17	3.94	0.18	0.40	0.77
	artifacts removed	2.45	9.47	0.26	0.54	1.15	1.20	4.18	0.18	0.39	0.77
	artifacts removed, updated	1.04	1.73	0.26	0.52	0.91	0.65	1.12	0.18	0.37	0.57
deppG-60s	whole database	2.87	15.59	0.04	0.08	0.49	1.24	6.21	0.04	0.06	0.25
	artifacts removed	2.87	15.59	0.05	0.08	0.49	1.24	6.21	0.04	0.06	0.25
	artifacts removed, updated	0.71	1.84	0.05	0.08	0.49	0.41	1.02	0.04	0.06	0.25

Table SI.2. The root mean square error (RMS) and mean absolute error (MAE) of the instantaneous respiratory rate (IRR) and instantaneous heart rate (IHR) estimation obtained by the deppG method for the Capnobase benchmark database in the following three scenarios. The estimated IRR and IHR are compared with a new reference IRR (respectively IHR) determined directly from the beginning of expirations in the capnogram signal (respectively the R-peaks of the electrocardiogram signal). Second, in the lines labeled “whole database” we provide results based on the whole database without removing any interval. Whereas in the lines labeled “artifacts removed”, we provide results when skipping labeled intervals containing artifacts provided in the database. Third, finally in the lines labeled “artifacts removed, updated”, we provide performance when we further skip intervals for which no beginning of expiration information are provided, in addition to skipping intervals labeled as containing artifacts. The unit of the HR is beats per minute, whereas the unit of RR is breaths per minute. Std: standard deviation. Q_1 and Q_3: first and third quartile.

reliable in all intervals. Clearly, for datasets containing artifacts, the newly generated reference curves tend to have wrong values which, in turn, impact negatively on the computed values of the proposed method’s performance. In particular, the datasets containing artifacts have high RMS and MAE when we use the new reference curves. This increases the statistical values used to measure the performance of deppG. The proposed method would have better performance in terms of RMS and MAE, if more reliable information on the R-peaks and expiration beginnings positions were available. This becomes more evident when we remove the intervals containing artifacts labeled in the database, “artifacts removed” case in Table SI.2, as well as the newly identified intervals with artifact in the capnometry signal mentioned before, “artifacts removed, updated” case in the same Table.

In summary, from Tables SI.1 and SI.2, we see that the best performance for the RR are obtained using the new reference curves and if we remove all the artifacts, including the ones not originally included in the database, whereas the performance for the HR are better when we compare the estimated curves with the given reference curves and after removing the artifacts.

1.2 ICASSP 2015 signal processing cup – PPG signal with motion

For each dataset, the ground-truth of heart rate, saved as variable ’BPM0’, is provided in the database and can be calculated from the simultaneously recorded ECG signal. Since our purpose is evaluating the IHR, it is important to detail how the reference AHR provided in the database is calculated [9]. In particular, the reference AHR is computed using a time window of $D = 8$ seconds in the following way. First, the number of cardiac cycles, denoted as H, is counted over time windows of length D (in seconds), the HR in each window is computed as $60 \times H/D$, which has unit beats per minute (BPM). Two successive time windows overlap by 6 seconds. Therefore, the first value in ’BPM0’ gives the calculated heart rate ground-truth in the first 8 seconds, while the second value in ’BPM0’ gives the calculated heart rate ground-truth from the 3rd second to the 10th second. The reference HR could thus be understood as the smoothed IHR by a zero-one window eight seconds in length, and this smoothing effect explains why we need to smooth out our estimated curves using Iterative Filtering (IF) [1, 2, 3], as explained previously, in order to obtain a good fitting result.

AAE	Sbj 1	Sbj 2	Sbj 3	Sbj 4	Sbj 5	Sbj 6	Sbj 7	Sbj 8	Sbj 9	Sbj 10	Sbj 11	Sbj 12	mean	std
TROIKA - Zhang *et al.* [9][ab]	2.87	2.75	1.91	2.25	1.69	3.16	1.72	1.83	1.58	4.00	1.96	3.33	2.42	0.78[c]
JOSS - Zhang [8][a]	1.33	1.75	1.47	1.48	0.69	1.32	0.71	0.56	0.49	3.81	0.78	1.04	1.29	0.90[c]
Khan *et al.* - double channel [6][a]	1.70	0.84	0.56	1.15	0.77	1.06	0.63	0.53	0.52	2.56	1.05	0.91	1.02	0.59[c]
Khan *et al.* - single channel [6][a]	1.77	1.94	0.73	1.19	0.51	1.09	0.52	0.43	0.36	3.43	0.89	0.98	1.15	0.88[c]
Khan *et al.* - double channel - 125 Hz [6][a]	1.83	0.85	0.63	1.21	0.65	1.03	0.70	0.50	0.47	2.83	1.14	0.90	1.06	0.67[c]
Temko - double channel [7][d]	1.23	1.26	0.72	0.98	0.75	0.91	0.67	0.91	0.54	2.61	0.94	0.98	1.04	0.54[c]
deppG - 125Hz[e] vs ref IHR	3.00	6.24	4.85	3.07	2.47	2.69	2.49	3.55	2.93	5.94	2.31	2.75	3.53	1.37
deppG-IF - 125Hz[f] vs ref AHR	1.29	0.55	0.50	0.67	0.47	0.75	0.68	0.52	0.30	3.32	0.52	0.60	0.85	0.81
deppG-8s - 125Hz[fa] vs ref AHR	1.31	0.90	0.77	0.93	0.75	0.86	0.75	0.77	0.67	3.28	0.70	0.80	1.04	0.73
deppG - 25Hz[g] vs ref IHR	2.80	5.42	4.84	3.37	2.58	2.71	2.61	3.52	2.91	6.37	2.31	2.80	3.52	1.31
deppG-IF - 25Hz[h] vs ref AHR	0.87	0.57	0.51	1.15	0.62	0.73	0.76	0.52	0.29	3.97	0.50	0.83	0.94	0.98
deppG-8s - 25Hz[ha] vs ref AHR	1.02	0.94	0.83	1.42	0.90	0.85	0.84	0.80	0.65	3.95	0.70	1.06	1.16	0.90

AAEP	Sbj 1	Sbj 2	Sbj 3	Sbj 4	Sbj 5	Sbj 6	Sbj 7	Sbj 8	Sbj 9	Sbj 10	Sbj 11	Sbj 12	mean	std
TROIKA - Zhang *et al.* [9][ab]	2.18	2.37	1.50	2.00	1.22	2.51	1.27	1.47	1.28	2.49	1.29	2.30	1.82	0.53[c]
JOSS - Zhang [8][a]	1.19	1.66	1.27	1.41	0.51	1.09	0.54	0.47	0.41	2.43	0.51	0.81	1.02	0.61[c]
deppG - 125Hz[e] vs ref IHR	2.85	6.01	4.06	2.66	1.89	2.32	2.01	3.23	2.61	3.96	1.60	2.16	2.95	1.23
deppG-IF - 125Hz[f] vs ref AHR	1.17	0.54	0.43	0.64	0.36	0.58	0.51	0.46	0.26	2.11	0.35	0.44	0.65	0.51
deppG-8s - 125Hz[fa] vs ref AHR	1.16	0.86	0.63	0.83	0.56	0.67	0.56	0.67	0.58	2.08	0.47	0.58	0.80	0.44
deppG - 25Hz[g] vs ref IHR	2.68	4.89	4.05	3.07	1.96	2.34	2.09	3.19	2.60	4.38	1.60	2.19	2.92	1.04
deppG-IF - 25Hz[h] vs ref AHR	0.83	0.56	0.44	1.24	0.50	0.56	0.56	0.46	0.25	2.74	0.33	0.64	0.76	0.67
deppG-8s - 25Hz[ha] vs ref AHR	0.93	0.90	0.68	1.42	0.70	0.67	0.62	0.70	0.57	2.71	0.47	0.80	0.93	0.61

Table SI.3. The average absolute error (AAE) and average absolute error percentage (AAEP) of the heart rate estimation for the ICASSP 2015 signal processing cup database, where the data is downsampled to 25 Hz instead of the original 125 Hz. The datasets have been also truncated as proposed in [8]. The unit for the heart rate is beats per minute. Sbj: subject. Std: standard deviation.

[a] 8s windows with 6s overlap

[b] Values reported in [8]

[c] This value has been recomputed using Matlab *std* function and does not match the value reported in the original paper.

[d] 8s windows with 6s overlap. It is not clear if in [7] the datasets are truncated or not. In fact, in that paper values are compared with both the TROIKA results for the untruncated database [9] and the JOint Sparse Spectrum (JOSS) ones related to the truncated one [8].

[e] $\lambda = 0.025$

[f] $\lambda = 0.023$

[g] $\lambda = 0.038$

[h] $\lambda = 0.048$

There is another important fact regarding the signal quality issue. Zhang in [8] proposed to truncate some of the datasets contained in the ICASSP Signal Processing Cup database in order to remove some motion artifacts. The excluded segments in that work were: the first 12 seconds of Set 2, the first 8 seconds of Set 3, the first 2 seconds of Set 4, the first 2 seconds of Set 8, the first 6 seconds of Set 10 and the first 2 seconds of Set 11. Furthermore, the same author proposed to downsample the PPG signals from 125 Hz to 25 Hz. We compare the performance of the deppG method when applied to truncated and downsampled signals in Table SI.3. From these results, the downsampling technique does not seem to help the deppG algorithm. In Table SI.3, we also show the AHR determined by the deppG-8s algorithm. We could see that the performance of the deppG-8s algorithm is slightly worse than the deppG-IF algorithm.

REFERENCES

1 .A. Cicone, J. Liu, and H. Zhou, *Adaptive local iterative filtering for signal decomposition and instantaneous frequency analysis*, Appl. Comput. Harmon. Anal. **41** (2016), no. 2, 384–411.

2 .______, *Hyperspectral chemical plume detection algorithms based on multidimensional iterative filtering decomposition*, Phil. Trans. R. Soc. A **374** (2016), 20150196.

3 .A. Cicone and H. Zhou, *Multidimensional iterative filtering method for the decomposition of high–dimensional non–stationary signals*, Numerical Mathematics: Theory, Methods and Applications **10** (2017), 278–298.

4 .C. Fischer, B. Domer, T. Wibmer, and T. Penzel, *An Algorithm for Real-Time Pulse Waveform Segmentation and Artifact Detection in Photoplethysmograms*, IEEE Journal of Biomedical and Health Informatics **2194** (2016), no. c, 1–1.

5 .N. Gambarotta, F. Aletti, G. Baselli, and M. Ferrario, *A review of methods for the signal quality assessment to improve reliability of heart rate and blood pressures derived parameters*, Med. Biol. Eng. Comput. **54** (2016), no. 7, 1025–1035.
6 .E. Khan, F. Al Hossain, S.Z. Uddin, S. Alam, and M. Hasan, *A Robust Heart Rate Monitoring Scheme Using Photoplethysmographic Signals Corrupted by Intense Motion Artifacts*, IEEE Trans. Biomed. Eng. **63** (2015), no. 3, 1–13.
7 .A. Temko, *Estimation of Heart Rate from Photoplethysmography during Physical Exercise using Wiener Filtering and the Phase Vocoder*, Conf Proc IEEE Eng Med Biol Soc., no. c, 2015, pp. 1500–3.
8 .Z. Zhang, *Photoplethysmography-based heart rate monitoring in physical activities via joint sparse spectrum reconstruction*, IEEE Trans. Biomed. Eng. **62** (2015), no. 8, 1902–1910.
9 .Z. Zhang, Z. Pi, and B. Liu, *TROIKA: A General Framework for Heart Rate Monitoring Using Wrist-Type Photoplethysmographic (PPG) Signals During Intensive Physical Exercise*, IEEE Trans. Biomed. Eng. **9294** (2014), no. c, 1–10.
